# Real-World Safety of COVID-19 mRNA Vaccines: A Systematic Review and Meta-Analysis

**DOI:** 10.3390/vaccines11061118

**Published:** 2023-06-19

**Authors:** Wanqian Xu, Weigang Ren, Tongxin Wu, Qin Wang, Mi Luo, Yongxiang Yi, Junwei Li

**Affiliations:** 1 School of Public Health, The Second Hospital of Nanjing, Nanjing Medical University, Nanjing 211166, China; wanqianxu@outlook.com (W.X.); lm56562021@163.com (M.L.); ian0126@126.com (Y.Y.); 2 The Clinical Infectious Disease Center of Nanjing, Nanjing 210003, China; rwg20161564@163.com (W.R.); wtx961002@163.com (T.W.); wangqin6621628@163.com (Q.W.); 3 Department of Infectious Diseases, The Second Hospital of Nanjing, Nanjing University of Chinese Medicine, Nanjing 210003, China

**Keywords:** COVID-19, SARS-CoV-2, mRNA vaccines, safety, observational study, meta-analysis

## Abstract

With the mass vaccination program for COVID-19 mRNA vaccines, there has been sufficient real-world study (RWS) on the topic to summarize their safety in the total population and in immunocompromised (IC) patients who were excluded from phase 3 clinical trials. We conducted a systematic review and meta-analysis to evaluate the safety of COVID-19 mRNA vaccines, with a total of 5,132,799 subjects from 122 articles. In the case of the total population vaccinated with first, second, and third doses, the pooled incidence of any adverse events (AEs) was 62.20%, 70.39%, and 58.60%; that of any local AEs was 52.03%, 47.99%, and 65.00%; that of any systemic AEs was 29.07%, 47.86%, and 32.71%. Among the immunocompromised patients, the pooled odds ratio of any AEs, any local AEs, and systemic AEs were slightly lower than or similar to those of the healthy controls at 0.60 (95% CI: 0.33–1.11), 0.19 (95% CI: 0.10–0.37), and 0.36 (95% CI: 0.25–0.54), with pooled incidences of 51.95%, 38.82%, and 31.00%, respectively. The spectrum of AEs associated with the vaccines was broad, but most AEs were transient, self-limiting, and mild to moderate. Moreover, younger adults, women, and people with prior SARS-CoV-2 infection were more likely to experience AEs.

## 1. Introduction

Coronavirus disease 2019 (COVID-19) is an acute respiratory disease caused by infection with the severe acute respiratory syndrome coronavirus 2 (SARS-CoV-2) virus, which has caused a global pandemic and public health crisis that rapidly gained momentum [1]. The COVID-19 pandemic has accelerated the employment of new vaccines at an unprecedented pace [2]. Of these, mRNA vaccines have emerged as a quick and efficient platform by which to address this challenging COVID-19 pandemic. In December 2020, the US Food and Drug Administration (FDA) issued emergency use authorizations (EUAs) for two mRNA vaccines, Pfizer/BioNTech’s BNT162b2 and Moderna’s mRNA-1273 [3]. Both mRNA vaccines showed high efficacy and mild to moderate adverse events (AEs) in phase 3 randomized clinical trials (RCTs) [4,5]. After that, they were widely applied in a real-world setting. Our World in Data subsequently reported that over 5.41 billion (69.7%) persons worldwide have received at least one dose of a COVID-19 vaccine [6]. However, mRNA vaccines, as an emerging technology, have the traits of rapid production and a short follow-up period, leading to public concerns about vaccine safety [7].

The present evidence regarding the safety of COVID-19 mRNA vaccines is based on phases 1–3 randomized controlled trials, observational studies, and the vaccine safety surveillance system. However, phase 3 clinical studies are constrained by sample size, the criteria needed for the inclusion of the population, and a tightly controlled setting that does not simulate the widespread distribution of the COVID-19 vaccines in the real world [8]. As the widespread vaccination programs progress, the scope of the vaccinated population steadily broadens to include elderly individuals, children/adolescents, pregnant and breastfeeding women, immunocompromised patients, etc. [9]. There are enough observational studies to offer data on the safety of COVID-19 mRNA vaccines in a wide population.

Currently, several systematic reviews and meta-analyses are available that have evaluated the safety of COVID-19 vaccines, based on RCTs [10,11,12,13,14,15], observational studies [13,15,16,17], or the vaccine safety surveillance system [13,16]. However, different COVID-19 vaccines were included in the studies, resulting in insufficient evidence regarding the safety evaluation of the COVID-19 mRNA vaccines. In addition, a heterogeneity analysis, which is lacking in some studies, needs to be conducted, since real-world studies require consideration of the complex factors regarding the population, vaccines, and study types. In addition, some studies [18,19,20,21,22,23] have shown the importance of the immunogenicity and efficacy of COVID-19 mRNA vaccines in immunocompromised patients but have not fully assessed their safety. Therefore, we conducted an independent and comprehensive study to assess the safety of COVID-19 mRNA vaccines, based on observational studies in the total population and immunocompromised patients, and explore the factors influencing safety.

## 2. Materials and Methods

### 2.1. Search Strategy

The meta-analysis of observational studies in epidemiology (MOOSE) guidelines were followed in the conduct of this study. We searched all publications related to COVID-19 mRNA vaccines in the following databases: PubMed, Web of Science, Scopus, and EMBASE. Three independent researchers searched for all papers released up until 25 June 2022, without any language restrictions. The keywords and MeSH terms used were “SARS-CoV-2”, “COVID-19”, “COVID-19 vaccines”, “mRNA vaccine”, “BNT162 vaccine”, “2019-nCoV vaccine mRNA-1273” and “safety”. The study protocol is registered on PROSPERO (CRD42022345197).

### 2.2. Eligibility Criteria

The study eligibility criteria were defined using the PICOS (population, intervention, comparator, outcome, and study) design approach. The eligible studies met the following specific criteria: (1) the study included observational studies of any design (cohort, case-control, and cross-sectional studies); (2) the COVID-19 mRNA vaccines included BNT162b2 (Pfizer-BioNTech (Pfizer Inc, New York, NY, USA)) or mRNA-1273 (Moderna (Moderna, Inc., Cambridge, MA, USA)), regardless of the dosage, schedule, preparation, or route of administration; (3) the study reported the safety data of the first, second or third dose of the vaccines, including the type, number, time of onset, duration, and severity of AEs.

The exclusion criteria were as follows: (1) the study did not use the SARS-CoV-2 mRNA vaccine as the exposure; (2) the study did not provide safety data for COVID-19 mRNA vaccines or only reported nonspecific outcomes; (3) the study types included RCT studies, animal experiments, in vitro studies, case reports, reviews, expert opinion, conference papers, editorials, preprints, letters, study protocols, news reports, and comments; (4) the study was a duplicate study or evaluated duplicate participants. 

### 2.3. Data Extraction

The following data were extracted from the qualifying studies: (1) basic information about the studies, including the first author, publication year, country, and study design; (2) characteristics of the study subjects, including the study population, sample sizes, age range, sex ratio, and history of prior SARS-CoV-2 infection; (3) the intervention measures, including the vaccine type, company, doses, concentration, route of administration, and injection interval; (4) the outcome measures, including the type, number, time of onset, duration, and severity of AEs. If defined data were unavailable, the required numbers were computed using the study’s percentages. Two researchers independently retrieved the following data from the selected studies, and the third researcher resolved the discrepancies.

### 2.4. Risk of Bias

The National Institutes of Health (NIH) (Bethesda, MD, USA) quality assessment tool [24] was used to evaluate the quality of the observational cohort and the cross-sectional studies. Those studies having scores of 11–14, 6–10, and 0–5 were classified as of good, fair, and poor quality, respectively. Moreover, the studies were assessed independently in terms of methodology by our study’s researchers, and any conflict of opinion was discussed or referred to a third researcher and was then resolved.

### 2.5. Statistical Analysis

We used the *I*^2^ statistics and Cochran’s Q-statistics to assess study heterogeneity. The *I*^2^ statistic was quantified as low (≤25%), moderate (25–50%), or high (>50%) and the significant heterogeneity was *p* < 0.10. When the *I*^2^ value was >50%, a random-effects model was used to calculate the overall results; otherwise, a fixed-effects model was used. To explore the sources of heterogeneity, we performed subgroup analysis according to study population (general population, healthcare workers, and patients), vaccine type (mRNA-1273, BNT162b2, and both), study type (cohort study and cross-sectional study), sample size (<100, 100–1000, 1000–10,000, and ≥10,000), survey time (within 7 days and more than 7 days) as grouping variables, while *p* < 0.05 in the Q test indicated significant differences between the subgroups. Sensitivity analysis was applied to assess the impact of the included studies by conducting a study-deletion analysis to establish whether any single study has a significant influence on the results. Egger’s test and a funnel plot were used to evaluate publication bias. In the presence of significant publication bias, the effect size was adjusted using the trim-and-fill method. All statistical analyses were conducted with R (version 4.1.0) and R Studio (version 1.4.1717) software (Posit Software, PBC formerly RStudio, PBC, Boston, MA, USA). In this meta-analysis, the value of *p* < 0.05 was considered statistically significant.

## 3. Results

### 3.1. Search Results and Study Characteristics

**Figure 1:**
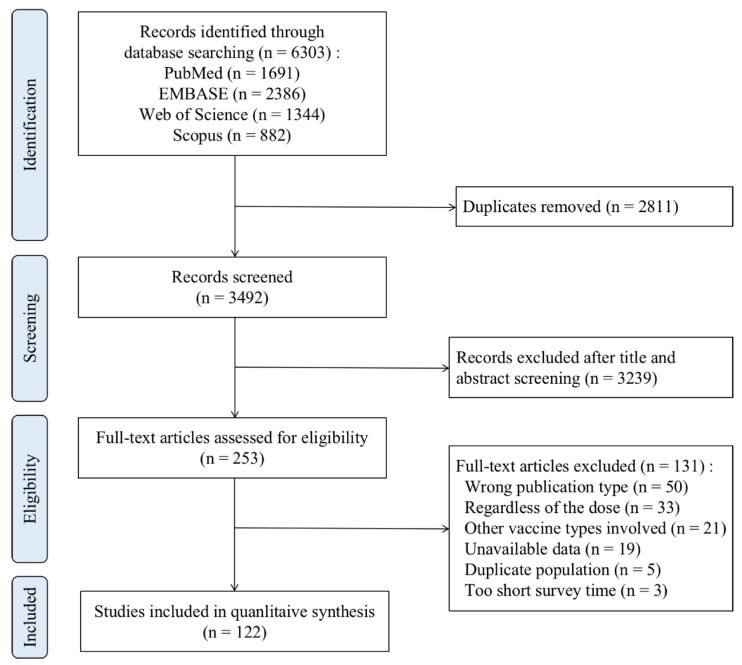
Flow diagram of the literature search and study selection for meta-analysis.

A total of 6303 publications were screened to investigate the safety of COVID-19 mRNA vaccines. According to the present criteria, 122 observational studies (415 datasets) were included in the meta-analysis (Figure 1). Studies assessing different vaccine types, doses, study populations, and control groups were included as separate meta-analysis datasets. A total of 5,132,799 study participants who had received COVID-19 mRNA vaccines were included in the safety set of the meta-analysis.

**Figure 2:**
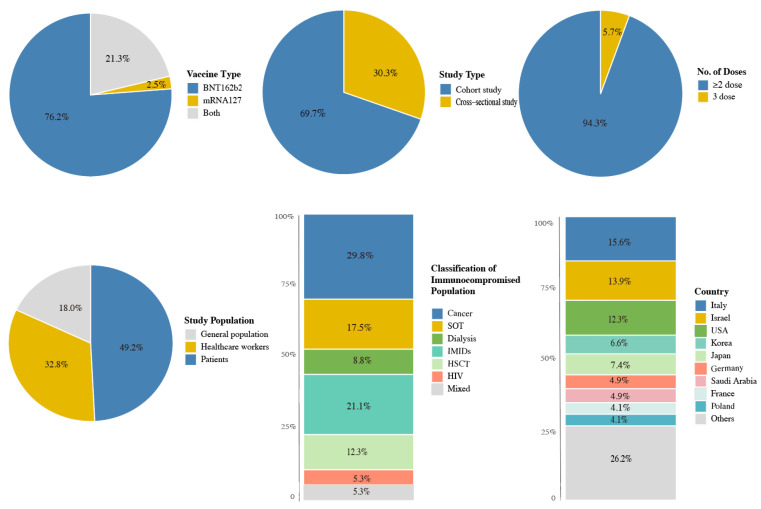
Distribution of included studies. Abbreviations: SOT, solid organ transplant; IMIDs, immune-mediated inflammatory diseases; HSCT, hematopoietic stem cell transplants; HIV, human immunodeficiency virus.

Out of 122 observational studies, 85 (69.7%) were cohort studies and 37 (30.3%) were cross-sectional studies. The number of studies on COVID-19 mRNA vaccines was 93 (76.2%) for BNT162b2, 3 (2.5%) for mRNA-1273, and 26 (21.3%) for both types. Regarding the number of doses, 115 studies (94.3%) involved first or/and second doses, and 7 (5.7%) involved third doses. Regarding the vaccine type, 22 studies (18.0%) evaluated the general population, 40 (32.8%) evaluated healthcare workers, and 60 (49.2%) evaluated the patients, of which 57 (95.0%) evaluated immunocompromised patients. Among them, 19 (15.6%) studies were conducted in Italy; 17 (13.9%) were in Israel; 15 (12.3%) were in the United States; 9 (7.4%) were in Japan; 8 (6.6%) were in Korea; 6 studies (4.9%) were in Germany and also in Saudi Arabia; 5 studies (4.1%) in France and also in Poland; fewer than 5 were in the other 17 countries (Figure 2). 

Regarding the quality assessment scores of the 122 studies, all studies were considered of fair quality (6–10 out of 14 possible points) per the NIH tool. The most frequent sources of bias were the lack of sample size calculation, the definition of the outcome measures or exposure levels, the blinding method for the subjects, the determination of the temporal order of exposure and outcome, and adjustment for potential confounding factors. 

### 3.2. Safety of COVID-19 mRNA Vaccines in the Total Population

**Figure 3:**
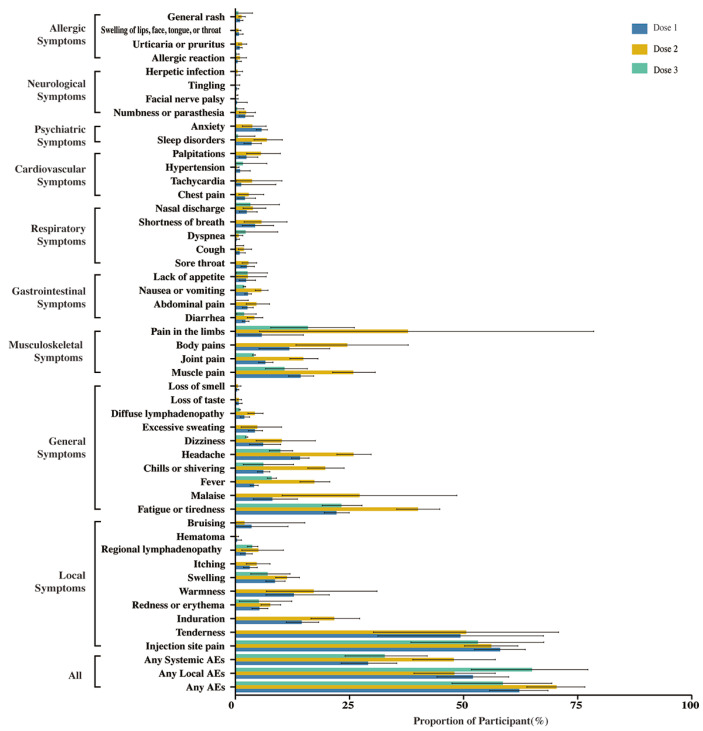
The incidence of adverse events related to the first, second, and third doses of COVID-19 mRNA vaccines in the total population. Abbreviation: AEs, adverse events.

The 50 outcome indicators of AEs were analyzed in the total population who had received the first, second, or third doses of COVID-19 mRNA vaccines (Figure 3). The pooled incidence of any AEs after vaccination was 62.20% (95% CI: 55.69–68.49%) for the first dose, 70.39% (95% CI: 63.84–76.56%) for the second dose, and 58.60% (95% CI: 47.46–69.33%) for the third dose. For any local AEs, the pooled incidence after vaccination was 52.03% (95% CI: 44.15–59.87%) for the first dose, 47.99% (95% CI: 39.12–56.92%) for the second dose, and 65.00% (95% CI: 51.72–77.21%) for the third dose. The common local AEs, in descending order of frequency, were injection site pain, tenderness, induration, warmness, swelling, and redness or erythema. Among any of the systemic AEs, the pooled incidence after vaccination was 29.07% (95% CI: 23.16–35.36%) for the first dose, 47.86% (95% CI: 38.86–56.94%) for the second dose, and 32.71% (95% CI: 24.02–42.02%) for the third dose. The systemic AEs were classified into 8 categories. The pooled incidences of the AEs with general systemic symptoms were high; the symptoms included fatigue or tiredness, malaise, headache, chills or shivering, fever, and dizziness, as well as musculoskeletal symptoms including pain in the limbs, muscle pain, body pain, and joint pain. The incidence of AEs in the other categories was less than 10%. It seems that the high pooled incidence of AEs was associated with the second vaccine dose; these included any AEs, any systemic AEs, and the most specific AEs. The third dose was associated with a high incidence of any local AEs. The symptoms usually peaked by days 1–3 after vaccination and disappeared within 7 days, and the severity was mild to moderate. 

The heterogeneity of the included studies was very high (*I*^2^ ≥ 50%) for most of the types of AEs. We subsequently conducted subgroup analyses to explore the sources of heterogeneity and the effects of heterogeneity on the AEs after the first or second vaccine dose within different populations and with different vaccine types, study types, sample sizes, and survey times. The subgroup analysis showed that the pooled incidence of AEs was lower in the group of patients and higher in people who had received mRNA-1273. There was no difference in the pooled incidence of AEs between the studies with different study types, sample sizes, and survey times (Table 1 and Table 2).

### 3.3. Safety of COVID-19 mRNA Vaccines in Immunocompromised Patients

**Figure 4:**
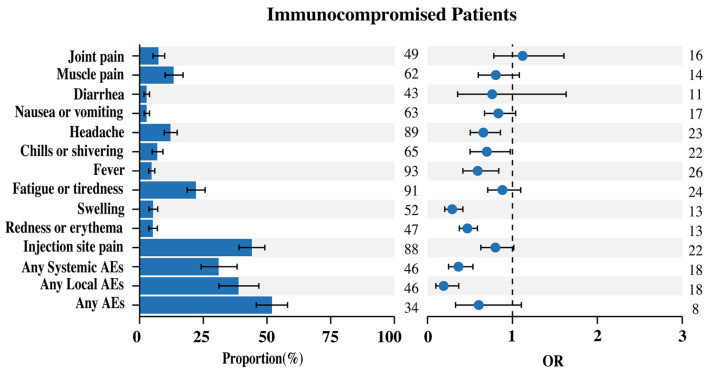
The incidence and OR of adverse events related to COVID-19 mRNA vaccines in immunocompromised patients. Abbreviations: AEs, adverse events; OR, odds ratio; CI, confidence interval.

**Figure 5:**
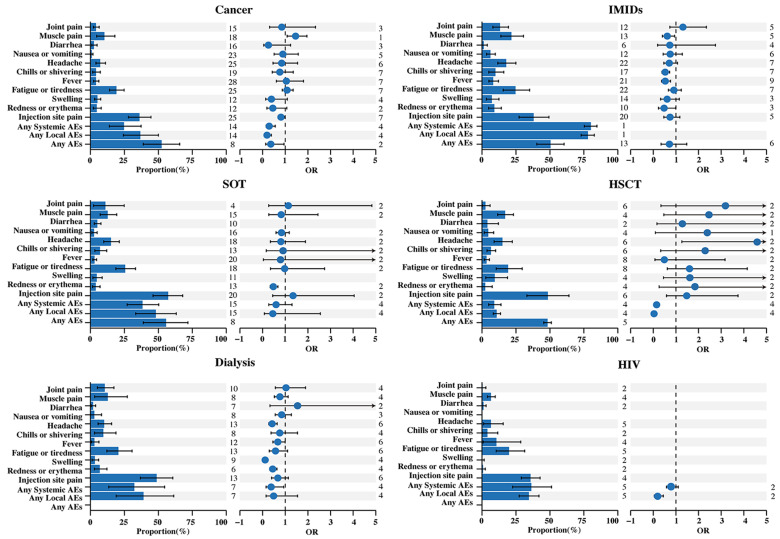
The incidence and OR of adverse events related to COVID-19 mRNA vaccines in immunocompromised patients with cancer, SOT, dialysis, IMIDs, HSCT, or HIV. Abbreviations: AEs, adverse events; OR, odds ratio; CI, confidence interval; SOT, solid organ transplant; IMIDs, immune-mediated inflammatory diseases; HSCT, hematopoietic stem cell transplants; HIV, human immunodeficiency virus.

We conducted the meta-analysis to evaluate the safety of COVID-19 mRNA vaccines in immunocompromised patients, using a total of 17,752 subjects from 57 articles. There were 4005 subjects from 17 articles showing patients with cancer, 2557 subjects from 10 articles showing patients with SOT, 779 subjects from 5 articles showing patients on dialysis, 5915 subjects from 12 articles showing patients with IMIDs, 485 subjects from 7 articles showing patients with HSCT, and 816 subjects from 3 articles showing patients with HIV. A total of 14 common AEs were analyzed in relation to the safety of immunocompromised patients. Among the immunocompromised patients, the pooled odds ratios (ORs) of any AEs, local AEs, and systemic AEs were slightly lower than or similar to those of the healthy controls at 0.60 (95% CI: 0.33–1.11), 0.19 (95% CI: 0.10–0.37), and 0.36 (95% CI: 0.25–0.54), with pooled incidences of 51.95% (95% CI: 45.80–58.07%), 38.82% (95% CI: 31.12–46.80%), and 31.00% (95% CI: 24.15–38.28%), respectively. Injection site pain (44.06%, 95% CI: 39.03–49.15%), fatigue or tiredness (22.10%, 95% CI: 18.66–25.73%), muscle pain (13.34%, 95% CI: 10.00–17.06%), and headache (12.09%, 95% CI: 9.66–14.74%) were the most common symptoms in the immunocompromised patients (Figure 4). We conducted a subgroup analysis based on the different disease types of the immunocompromised patients (Figure 5).

Among the cancer patients, the pooled incidence of any AEs, any local AEs, and any systemic AEs were 52.40% (95% CI: 38.92–65.72%), 36.67% (95% CI: 24.26–50.00%), and 24.71% (95% CI: 13.95–37.24%), respectively, after any dose. Injection site pain (36.09%, 95% CI: 28.00–44.59%), fatigue or tiredness (19.13%, 95% CI: 14.02–24.80%), and muscle pain (10.31%, 95% CI: 4.54–17.94%) were the most common symptoms. Pooled analyses of the AEs showed a decreased and statistically significant risk in the cancer patients compared with the healthy controls for any AEs (OR: 0.36, 95% CI: 0.14–0.95), any local AEs (OR: 0.19, 95% CI: 0.10–0.39), any systemic AEs (OR: 0.29, 95% CI: 0.15–0.56), and injection site pain (OR: 0.81, 95% CI: 0.68–0.97). The risk of muscle pain (OR: 1.46, 95% CI: 1.08–1.96) was significantly increased, but the other AEs were not statistically significant (Figure 5).

Among the SOT patients, the pooled incidence of any AEs, any local AEs, and any systemic AEs were 55.68% (95% CI: 38.94–71.79%), 48.18% (95% CI: 33.31–63.22%), and 38.34% (95% CI: 27.06–50.29%), respectively, after any dose. Injection site pain (57.19%, 95% CI: 46.09–67.93%), fatigue or tiredness (25.61%, 95% CI: 18.67–33.20%), headache (15.11%, 95% CI: 9.83–21.24%), and muscle pain (12.69%, 95% CI: 7.21–19.38%) were the most common symptoms. Only redness or erythema (OR: 0.47, 95% CI: 0.33–0.68) showed a decreased and statistically significant risk in the SOT patients compared with the healthy controls (Figure 5).

Among the dialysis patients, the pooled incidence of any local AEs and any systemic AEs were 39.08% (95% CI: 18.92–61.35%) and 32.30% (95% CI: 13.51–54.60%), respectively, after any dose. Injection site pain (48.69%, 95% CI: 36.77–60.68%), fatigue or tiredness (20.58%, 95% CI: 12.05–30.62%), and muscle pain (12.78%, 95% CI: 3.13–27.12%) were the most common symptoms. Any systemic AEs (OR: 0.38, 95% CI: 0.15–0.94), redness or erythema (OR: 0.45, 95% CI: 0.31–0.63), swelling (OR: 0.11, 95% CI: 0.06–0.22), fever (OR: 0.67, 95% CI: 0.45–0.99), and headache (OR: 0.42, 95% CI: 0.27–0.64) showed a decreased and statistically significant risk in the dialysis patients compared with the healthy controls (Figure 5).

Among the IMID patients, the pooled incidence of any AEs, any local AEs, and any systemic AEs were 50.40% (95% CI: 40.39–60.40%), 78.01% (95% CI: 72.98–82.67%), and 80.14% (95% CI: 75.27–84.60%), respectively, after any dose. Injection site pain (38.01%, 95% CI: 27.31–49.32%), fatigue or tiredness (24.68%, 95% CI: 15.59–35.04%), muscle pain (21.65%, 95% CI: 13.91–30.52%), and headache (17.78%, 95% CI: 11.58–24.93%) were the most common symptoms. Redness or erythema (OR: 0.48, 95% CI: 0.23–0.99), fever (OR: 0.53, 95% CI: 0.37–0.76), chills or shivering (OR: 0.53, 95% CI: 0.39–0.72), and muscle pain (OR: 0.62, 95% CI: 0.40–0.94) showed a decreased and statistically significant risk in the IMID patients compared with the healthy controls (Figure 5).

Among the HSCT patients, the pooled incidence of any AEs, any local AEs, and any systemic AEs were 48.21% (95% CI: 45.41–51.01%), 10.81% (8.26%, 95% CI: 13.65%), and 8.94% (4.91%, 95% CI: 13.97%), respectively, after any dose. Injection site pain (48.44%, 95% CI: 33.03–63.99%), fatigue or tiredness (19.17%, 10.48–29.58%), muscle pain (16.96%, 95% CI: 11.51–23.14%), and headache (15.03%, 95% CI: 8.91–22.29%) were the most common symptoms. Any local AEs (OR: 0.03, 95% CI: 0.03–0.05) and any systemic AEs (OR: 0.15, 95% CI: 0.11–0.20) showed a decreased and statistically significant risk, while headache (OR: 7.39, 95% CI: 1.26–43.3) was significantly increased in the HSCT patients compared with the healthy controls (Figure 5).

Among the HIV patients, the pooled incidence of any local AEs and any systemic AEs were 34.47% (95% CI: 27.36–41.94%) and 36.25% (95% CI: 22.53–51.19%), respectively, after any dose. Injection site pain (35.70%, 95% CI: 28.96–42.73%), fatigue or tiredness (19.86%, 95% CI: 10.41–31.40%), and fever (10.59%, 95% CI: 0.86–28.51%) were the most common symptoms. Any local AEs (OR: 0.14, 95% CI: 0.04–0.44) showed a decreased and statistically significant risk in the HIV patients compared with the healthy controls (Figure 5).

### 3.4. The Influencing Factors on the Safety of COVID-19 mRNA Vaccines

A total of 14 AEs were included in the analysis of influencing factors on the COVID-19 mRNA vaccine safety. We found that those individuals characterized by female sex, younger age, and prior SARS-CoV-2 infection had a higher risk of AEs.

**Figure 6:**
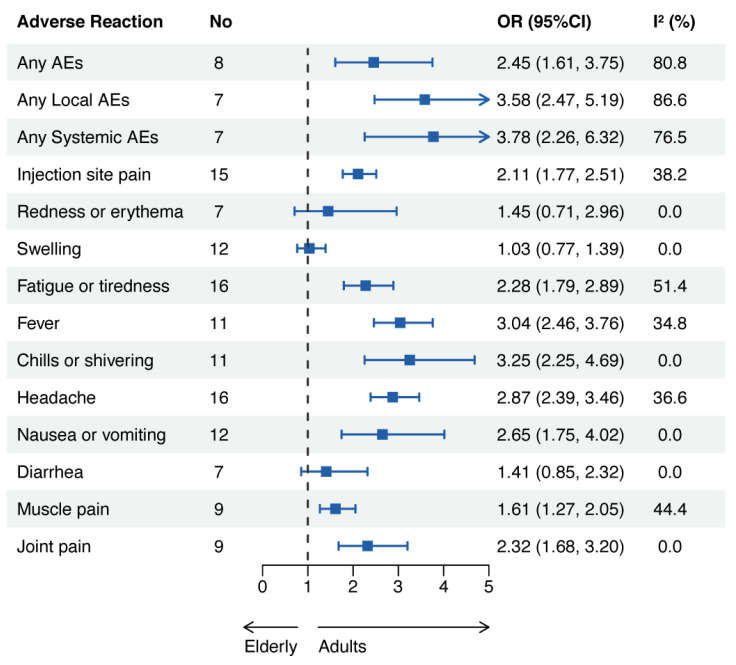
Adverse events related to COVID-19 mRNA vaccines in adults and the elderly. Abbreviations: AEs, adverse events; OR, odds ratio; CI, confidence interval.

Most AEs were significantly higher in the adult group than in the elderly group, except for redness or erythema, swelling, or diarrhea. The pooled OR of any AEs was 2.45 (95% CI: 1.61–3.75), that of any local AEs was 3.58 (95% CI: 2.47–5.19), and that of any systemic AEs was 3.78 (95% CI: 2.26–6.32) (Figure 6).

**Figure 7:**
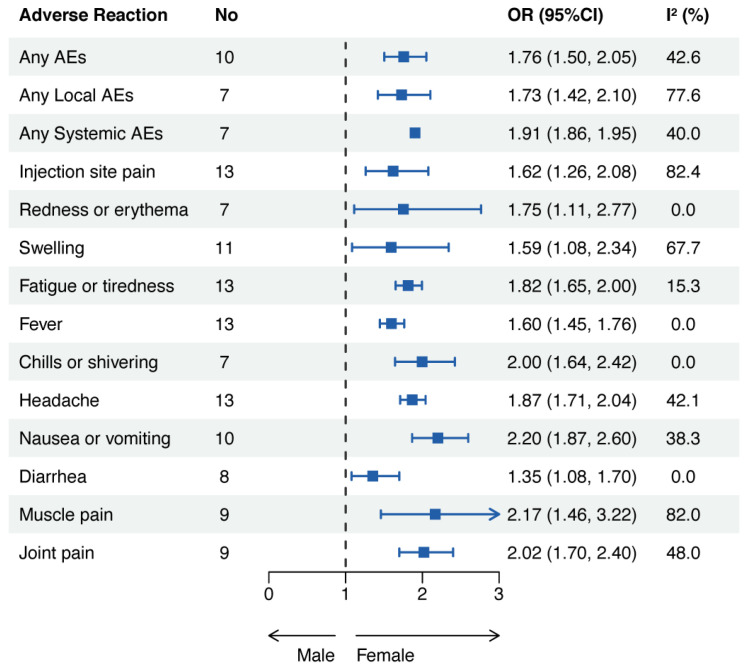
Adverse events related to COVID-19 mRNA vaccines in females and males. Abbreviations: AEs, adverse events; OR, odds ratio; CI, confidence interval.

All 14 AEs were statistically significant, suggesting that the pooled incidence of AEs was significantly higher in the female group than in the male group. The pooled OR of any AEs was 1.76 (95% CI: 1.50–2.05), any local AEs OR 1.73 (95% CI: 1.42–2.10), and any systemic AEs OR 1.91 (95% CI: 1.86–1.95) (Figure 7).

**Figure 8:**
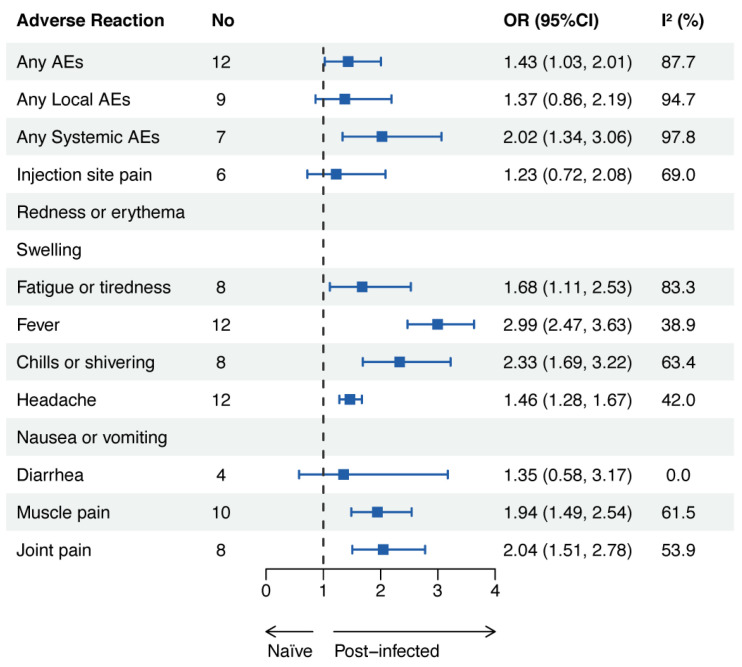
Adverse events related to COVID-19 mRNA vaccines in the population with and without prior SARS-CoV-2 infection. Abbreviations: AEs, adverse events; OR, odds ratio; CI, confidence interval.

The participants who had a prior SARS-CoV-2 infection showed statistical significance for most AEs but not for any local AEs, injection site pain, or diarrhea. The pooled OR of any total AEs was 1.43 (95% CI: 1.03–2.01), that of any local AEs was 1.37 (95% CI: 0.86–2.19), and that of any systemic AEs was 2.02 (95% CI: 1.34–3.06) (Figure 8).

### 3.5. Sensitivity Analysis and Publication Bias

We performed a sensitivity analysis by omitting one study at a time. The results indicated that our study was stable. Funnel plot analysis and an Egger’s test were used to examine the significance of the publication bias underlying this study. Then, we applied the trim-and-fill method to adjust for publication bias. The adjusted results of the pooled analysis were similar to those of the original analysis in terms of safety evaluation.

## 4. Discussion

COVID-19 continues to pose a threat to the world’s public health, even though it has been over two years since the initial cases were reported. The high speed of mRNA vaccine development and the negative reports based on individual results have deepened concerns and vaccine hesitation among the public, which might impede widespread immunization [25]. Therefore, our study provides a comprehensive evaluation of the existing observational studies on the safety of COVID-19 mRNA vaccines in the wider population.

In our study of the total population, any AEs (62.2%, 70.39%, 58.60%), any local AEs (52.03%, 47.99%, 65.00%), and any systemic AEs (29.07%, 47.86%, 32.71%) were the primary endpoints for safety, after the first, second, and third doses of vaccines, respectively. The pooled incidence of AEs from the observational studies was lower than those from the clinical trials [4,5] and higher than those from the vaccine safety surveillance systems [13]. It seems that the second dose was associated with a higher pooled incidence of AEs, but the third dose was associated with prominent local AEs. Moreover, the types of specific local AEs and systemic AEs were consistent with the clinical trials [4,5], but the spectrum of AEs associated with the vaccines was broader than those specified; most AEs were transient, self-limiting, and mild to moderate.

Our study provides evidence in support of the safety of COVID-19 mRNA vaccines based on observational studies, and this meta-analysis enabled a broad representation of the population at large, a longer timespan from development to application, and a wide range of AEs. In the early clinical trials, children/adolescents, pregnant and breastfeeding women, elderly individuals with comorbidities, and immunocompromised patients were excluded [4,5]. Thus, the results of the trials may differ from results in the real world, but the trials may be constrained by the sample size, the inclusion population criteria, and the tightly controlled setting. Moreover, spontaneous (or passive) immunization safety surveillance systems, such as the vaccine adverse event reporting system (VAERS), could continuously and openly identify more uncommon and rarer AEs [13]. However, after vaccination, many participants did not report their AEs to VAERS, resulting in a lower pooled incidence rate than with AEs reported by those in the clinical trials. In brief, our study is potentially much more generalizable and representative.

Real-world research inevitably results in high heterogeneity because of the complex factors of the population, vaccine, and study type. Therefore, subgroup analyses were conducted to assess the cause of this heterogeneity by evaluating the different populations, vaccine types, study types, sample sizes, and survey times. The research results showed that the patients had a lower pooled incidence of AEs than in the general population and healthcare workers, which set the stage for our subsequent study of immunocompromised patients (95% of the patients). In addition, our findings were consistent with clinical studies illustrating that mRNA-1273 was associated with higher AEs than BNT162b2 [4,5]. Both BNT162b2 and mRNA-1273 are lipid nanoparticles (LNPs)-formulated mRNA vaccines. LNPs are negatively charged mRNA delivery vehicles composed of four lipid components, namely, an ionizable cationic lipid, phospholipids, cholesterol, and lipid-linked polyethylene glycol (PEG). Of these, the ionizable cationic lipid plays an important role in aiding intracellular delivery and endosomal escape [26]. Previous studies have shown that the ionizable cationic lipid has an impact on the local reactogenicity and cytokine expression with parenteral administration and is a critical parameter for providing adjuvant activity to LNPs [27]. The difference between the safety of BNT162b2 and mRNA-1273 vaccinations may come from the type and composition of an mRNA formulation, especially LNPs.

Our study revealed that immunocompromised patients had a slightly lower or similar pooled incidence of AEs than healthy controls. Injection site pain, fatigue or tiredness, muscle pain, and headache were the most common symptoms in immunocompromised patients with any kind of disease type. Current research shows that immunocompromised patients, particularly transplant recipients, developed reduced immunogenicity following two doses of the COVID-19 mRNA vaccine [18]. The diminished immunogenicity of COVID-19 mRNA vaccines in immunocompromised patients might be related to the low incidence of AEs. Due to the increased risk of severe COVID-19 illness and death in moderately or severely immunocompromised patients, the CDC recommended that these patients receive an updated COVID-19 booster to protect against severe COVID-19 outcomes [28]. In addition, the COVID-19 mRNA vaccine safety of patients with HIV and HSCT needs more research, as shown by the insufficient data in our study.

The impact of individual factors on AEs caused us concern. Several meta-analyses showed that the pooled incidence of AEs after COVID-19 vaccination (including COVID-19 mRNA vaccines) was higher in elderly individuals than in adults; these meta-analyses were based on clinical studies [12,16,29] or VAERS [13]. Our study was derived from a large number of observational studies, strengthening this point of view for COVID-19 mRNA vaccines. In addition, in terms of the four vaccines that are currently recommended for the elderly, including vaccines for influenza, herpes zoster, pneumococcal disease, and tetanus and diphtheria, it was reported that the immune response to vaccination was reduced in the elderly compared with young, healthy adults [30,31,32]. The reason for this is that immunosenescence leads to a lower risk of AEs and immunogenicity after vaccination in elderly individuals [33,34,35]. This could be due to the function of the immune system’s declining with age and because both the inflammatory response and the protective immune response are slower and weaker in elderly individuals than in adults. However, the current evidence was insufficient to suggest whether older age means safer vaccination because elderly people with underlying diseases are at a high risk of developing critical illness or death. Since the elderly are susceptible to SARS-CoV-2 infection, further methods could be taken, such as additional vaccination doses, the use of adjuvants, and alternative routes of immunization, to improve the efficacy and immunogenicity of vaccines, which should be considered in conjunction with safety [36].

Our research indicated that women were at higher risk of AEs than men. The different incidences of AEs between females and males were related to variations in the immunogenicity to vaccination [37]. Other studies showed that women vaccinated with the influenza vaccine or infected with SARS-CoV-2 also had more AEs and stronger immune responses than men [38,39]. The sex differences in AEs are typically regarded as complex, involving both biological and behavioral variables, despite a few arguments supporting an association with hormone differences [40]. Therefore, more care should be taken to monitor the AEs of vaccinated women. In the future, a gender-specific vaccination strategy could be considered due to the more responsive immune system seen in females.

It was reported in our study that individuals with prior SARS-CoV-2 infection had more AEs than those without a history of COVID-19 and that these patients were especially prone to systemic symptoms such as fever and chills or shivering. The participants with past infections had a higher antibody titer than those without prior SARS-CoV-2 infections after vaccination [9]. This increased reactogenicity relates to increased immunogenicity. The available evidence supports the proposition of a single-dose immunization for people with prior SARS-CoV-2 infection.

Our meta-analysis still has several limitations. First, the included cohort studies were cross-sectional in nature. Thus, one of the reasons for fair-quality evidence was that there was no control set of studies, which inevitably led to high heterogeneity in the incidence of AEs. We took multiple approaches to address this problem in single-arm studies, such as transformation and the correction methods of single rates (PFT, PAS, PRAW, PLN, and PLOGIT), a pooling method using a random-effects model, and subgroup analysis. After statistical correction and analysis, the heterogeneity of the pooled incidence of AEs was still high. We speculated that the heterogeneity stemmed from the data itself, possibly owing to the diversity of age, gender, ethnicity, geography, and other factors beyond our control. Second, we estimated the pooled odds ratio (OR) and 95% confidence interval (CI) for the risk of AEs in immunocompromised patients with different disease types. Due to the limited number and limited sample size of the included studies, the results from the HIV and HSCT patients were not stable and were for reference only. The data regarding AEs were self-reported and were, thus, subject to recall and reporting bias. In addition, we cannot provide sufficient evidence of serious AEs and long-term safety due to the limitations of the original studies’ designs.

## 5. Conclusions

In conclusion, the AEs reported after COVID-19 mRNA vaccines were transient, self-limiting, and mild to moderate, according to real-world observational studies. These vaccines were still safe for use in immunocompromised populations. It seems that the high pooled incidence of any AEs and any systemic AEs was associated with the second dose, and the third dose was associated with a high incidence of any local AEs. Younger adults, women, and people with prior SARS-CoV-2 infection were more likely to experience negative AEs. The results provided clear data-driven evidence to support the safety of COVID-19 mRNA vaccines for the public and policymakers. However, more information about rare and serious AEs should be evaluated throughout a long period of surveillance.

## Figures and Tables

**Table 1:** Subgroup analysis of the total AEs, local AEs, and systemic AEs after the first dose of the COVID-19 mRNA vaccine. Description: Subgroups according to study populations, vaccine types, study types, survey times, and population sizes. Abbreviations: AEs, adverse events; CI, confidence interval.

Group	No	Any AEs Proportion (95% CI)	*p*-Value	No	Any Local AEs Proportion (95% CI)	*p*-Value	No	Any Systemic AEs Proportion (95% CI)	*p*-Value
Overall	52	0.62 (0.56–0.68)		43	0.52 (0.44–0.60)		40	0.29 (0.23–0.35)	
Study population			0.0054			0.0258			0.0197
General population	11	0.68 (0.55–0.80)		8	0.63 (0.44–0.80)		7	0.27 (0.16–0.40)	
Healthcare workers	26	0.67 (0.58–0.76)		14	0.61 (0.54–0.68)		12	0.40 (0.32–0.48)	
Patients	15	0.48 (0.39–0.57)		21	0.42 (0.30–0.54)		21	0.23 (0.15–0.33)	
Vaccine type			0.6097			<0.0001			<0.0001
BNT162b2	43	0.61 (0.53–0.68)		35	0.45 (0.37–0.53)		32	0.24 (0.18–0.30)	
mRNA-1273	3	0.71 (0.41–0.93)		5	0.83 (0.70–0.93)		5	0.58 (0.51–0.65)	
Both	6	0.67 (0.54–0.79)		3	0.76 (0.65–0.86)		3	0.44 (0.23–0.67)	
Study type			0.0585			0.9568			0.3025
Cohort study	28	0.56 (0.49–0.63)		36	0.52 (0.43–0.61)		34	0.28 (0.21–0.35)	
Cross-sectional study	24	0.69 (0.58–0.79)		7	0.52 (0.40–0.65)		6	0.36 (0.23–0.49)	
Survey time			0.004			0.0083			0.0625
Within 7 days	14	0.58 (0.45–0.71)		17	0.44 (0.29–0.59)		16	0.39 (0.29–0.49)	
More than 7 days	22	0.55 (0.45–0.65)		17	0.43 (0.30–0.56)		16	0.24 (0.16–0.32)	
No description	16	0.75 (0.67–0.83)		9	0.65 (0.55–0.75)		8	0.21 (0.11–0.33)	
Population size			0.0772			0.0645			0.0835
<100	10	0.64 (0.51–0.75)		10	0.67 (0.53–0.81)		9	0.40 (0.31–0.50)	
100–1000	31	0.62 (0.53–0.71)		21	0.49 (0.37–0.61)		19	0.25 (0.17–0.35)	
1000–10,000	9	0.64 (0.46–0.80)		9	0.40 (0.28–0.54)		9	0.25 (0.14–0.38)	
≥10,000	2	0.46 (0.33–0.59)		3	0.63 (0.52–0.74)		3	0.37 (0.14–0.64)	

**Table 2:** Subgroup analysis of total AEs, local AEs, and systemic AEs after the second dose of the COVID-19 mRNA vaccine. Description: Subgroups according to study populations, vaccine types, study types, survey times, and population sizes. Abbreviations: AEs, adverse events; CI, confidence interval.

Group	No	Any AEs Proportion (95% CI)	*p*-Value	No	Any Local AEs Proportion (95% CI)	*p*-Value	No	Any Systemic AEs Proportion (95% CI)	*p*-Value
Overall	54	0.70 (0.64–0.77)		43	0.48 (0.39–0.57)		40	0.48 (0.39–0.57)	
Study population			<0.0001			0.0041			0.0169
General population	13	0.82 (0.74–0.89)		7	0.65 (0.43–0.83)		6	0.59 (0.30–0.85)	
Healthcare workers	24	0.77 (0.68–0.84)		13	0.61 (0.48–0.74)		11	0.63 (0.49–0.75)	
Patients	17	0.50 (0.39–0.61)		23	0.35 (0.25–0.46)		23	0.38 (0.28–0.49)	
Vaccine type			0.0262			0.011			0.0003
BNT162b2	43	0.70 (0.62–0.77)		34	0.42 (0.33–0.51)		31	0.41 (0.32–0.51)	
mRNA-1273	4	0.86 (0.74–0.94)		5	0.75 (0.49–0.95)		5	0.78 (0.63–0.90)	
Both	7	0.65 (0.54–0.76)		4	0.65 (0.47–0.80)		4	0.59 (0.40–0.77)	
Study type			0.0766			0.3825			0.0714
Cohort study	32	0.65 (0.57–0.73)		37	0.46 (0.37–0.56)		35	0.46 (0.36–0.56)	
Cross-sectional study	22	0.77 (0.67–0.86)		6	0.58 (0.35–0.79)		5	0.62 (0.47–0.76)	
Survey time			0.022			0.0515			0.3133
Within 7 days	14	0.69 (0.57–0.80)		18	0.50 (0.48–0.71)		17	0.55 (0.43–0.67)	
More than 7 days	24	0.63 (0.52–0.73)		17	0.42 (0.28–0.57)		16	0.44 (0.28–0.60)	
No description	16	0.82 (0.73–0.90)		8	0.34 (0.15–0.56)		7	0.39 (0.19–0.61)	
Population size			0.1258			0.1601			0.0856
<100	10	0.69 (0.51–0.85)		10	0.65 (0.46–0.81)		9	0.69 (0.50–0.85)	
100–1000	34	0.68 (0.59–0.76)		22	0.41 (0.29–0.54)		20	0.40 (0.28–0.52)	
1000–10,000	8	0.82 (0.76–0.88)		8	0.44 (0.27–0.61)		8	0.45 (0.27–0.63)	
≥10,000	2	0.62 (0.44–0.78)		3	0.60 (0.31–0.85)		3	0.53 (0.22–0.83)	

## Data Availability

Additional data related to this paper may be requested from the authors.
